# Depression in the schistosomiasis japonica population based on the PHQ-9 scale: a cross-sectional survey from Jiangxi Province, China

**DOI:** 10.1038/s41598-024-74510-5

**Published:** 2024-10-17

**Authors:** Aizhen Hu, Dele Liu, Huiqun Xie, Xia Wu, Kexing Liu, Xuyun Zhang, Linlin Li, Xing Zhou, Fei Hu

**Affiliations:** 1 Jiangxi Provincial People’s Hospital, The First Affiliated Hospital of Nanchang Medical College, Nanchang, 330006 People’s Republic of China; 2 Jiangxi Provincial Institute of Parasitic Diseases, Nanchang, 330096 People’s Republic of China; 3 Yushan Schistosomiasis Control Station, Yushan, 334000 People’s Republic of China

**Keywords:** Depression, Patient health questionnaire, Diagnostic interview, Schistosomiasis, Mental health, Jiangxi Province, Preventive medicine, Psychology

## Abstract

Depression is the second leading-cause of disability in China. Although studies have shown that more than 80% of patients with advanced schistosomiasis (AS) suffer from anxiety and depression, these study groups are all hospitalized patients with AS and do not represent the extent of the disease in the whole group. To our knowledge, there are no studies assessing the severity of depression in patients with other forms of schistosomiasis japonicum. Therefore, it is necessary to evaluate the occurrence and potential risk factors of depression in the schistosomiasis endemic population. This cross-sectional study was conducted in Jiangxi Province, where schistosomiasis is relatively common in China, as the investigation site. The Patient Health Questionnaire-9 (PHQ-9) scale was selected to assess the depressive symptoms in the study population. At the same time, basic personal information of the research subjects and relevant socio-economics and schistosomiasis endemic area indicators were collected. The survey results show that AS has the highest incidence of depression (34.35%), while non-advanced schistosomiasis (N-AS) and control group (CG) have 22.35% and 22.24% respectively; the incidence of depression in AS is significantly higher than in N-AS and CG, while there is no statistical significance in the comparison between N-AS and CG; the incidence of mild depressive symptoms accounts for 61.08%-75.54% of the total incidence of depression in different groups; multivariate analysis shows that the occurrence of moderate/severe depressive symptoms in the AS group was significantly related to above 60 years old, male, the combination of other serious diseases, personal financial difficulties, and marshland and lake endemic areas. In the N-AS group, the occurrence of moderate/severe depressive symptoms was significantly related to the combination of other serious diseases, personal financial difficulties, significant correlation between marshland and lake endemic areas and the level of control of schistosomiasis epidemics. In conclusion, depression is still relatively common among patients with schistosomiasis patients, especially those with AS. It is recommended that the government and relevant departments consider mental health care when developing prevention and control work in schistosomiasis-endemic areas, in order to effectively protect the physical and mental health of schistosomiasis patients and residents in endemic areas.

## Introduction

Depression is one of the most common mental illnesses in the world, with the highest suicide rate. It brings suffering to the patients and their families, and its damage to society is incomparable to other diseases. According to the World Health Organization, more than 300 million people worldwide suffer from depression^1^. In the worst cases, depressed people may commit suicide^2^. According to the World Health Organization (WHO), depression will be the second leading cause of disability by 2020, and the first by 2040!

Depression as a psychological state is largely overlooked^3,4^, and people with depression are often underdiagnosed and/or misdiagnosed, which hinders effective treatment^5–7^. Approximately 4.7% of the global population have experienced a depressive episode in any 12 months, with the average episode lasting approximately six months^8^. In China, depression is the second leading-cause of disability^9^. The Global Burden of Disease Study 2019 (GBD2019) showed that the number of cases of depression in China was 5,056,200, with a prevalence rate of 3.52%. The weighted-adjusted calculation in the China Mental Health Survey showed that the weighted lifetime prevalence of adult depressive disorders in China was 6.8% (*95% CI*:5.8–7.8), of which the weighted major depressive disorder was 3.4% (*95% CI*:2.9–3.9)^10^. According to The Blue Book of 2022 National Depression, there are currently 95 million people suffering from depression in China, and approximately 280,000 people commit suicide each year, 40% of whom suffer from depression. A meta-analysis conducted by Gu et al. in 2013 showed that the current overall estimate of the prevalence of major depression in China is 1.6%^11^. Some of the survey literature for the period 2002–2007 pointed out that there were regional differences in the prevalence of depression among rural groups in different provinces and cities in China, such as 1.10% in Jiangxi Province, 2.10% in Liaoning Province, 2.36% in Zhejiang Province, 2.79% in Hebei Province, 3.67% in Qingdao City of Shandong Province, 4.0% in Liuyang City of Hunan Province, and 4.19% in Beijing City^12–18^.

Schistosomiasis is an endemic parasitic disease caused by infection with *Schistosoma*, which is endemic in many countries and regions of the world, with approximately 779 million people at risk of infection^19^. Only Schistosomiasis japonica is endemic in China, and it is distributed in 12 provinces, municipalities and autonomous regions south of the Yangtze River in China, with the threatened population living mainly in rural areas. *Schistosoma* eggs can be deposited in the liver, intestines, and other tissues and organs of the patient, resulting in a decline in the ability to work and functional disability in some patients, seriously affecting the quality of their survival and the prognosis of the disease^20^. Previous studies have shown that more than 80% of patients with advanced schistosomiasis (AS) suffer from anxiety and depression^21^. One researcher conducted a meta-analysis using Chinese and English literature published from 2000 to 2022 and found that the co-morbidity between AS and depression was 61% *(95% CI*:48%-72%)^22^. This suggests that the occurrence of depression is particularly severe in the AS group. While there is no relevant literature on the severity of the occurrence of depression in groups who have been infected with schistosomes but whose disease course has not progressed to AS, there is no evidence of increased vulnerability to depressive symptoms in people with schistosomiasis. There are also no evidence-based interventions exist. Therefore, there is a need to assess the occurrence of depression and potential risk factors in schistosomiasis populations.

The Patient Health Questionnaire-9 (PHQ-9) is a scale developed by the National Institute of Mental Health (NIMH) to assess depressive symptoms. It consists of nine questions, each corresponding to a different aspect of depressive symptoms, including mood, sleep, appetite, and attention. Each question has four response options corresponding to different levels of symptom frequency, ranging from “hardly ever” to "almost every day". The PHQ-9 scale is a simple, validated depression screening tool designed to screen and assess the severity of depressive symptoms in both clinical and research settings. assess the severity of depressive symptoms in clinical and research settings. It is commonly used as a unidimensional measure of depressive symptoms because it identifies, differentiates, and monitors depressive symptoms and is easy to administer good reliability and validity^23–25^. It is now widely used to screen for depression in primary care populations^26–28^, in patient populations with a variety of medical conditions^29–32^, and in multicultural populations^33,34^. Since the application of the PHQ-9, researchers have demonstrated reasonable validity and reliability in identifying depression in a variety of settings^35–39^. However, it should be emphasized that the results of the PHQ-9 measurement are only a reference, and the final diagnosis and treatment plan must be determined by a professional doctor based on the actual situation.

In China, the National Health Commission of the People’s Republic of China issued the "Work Program for Exploring Specialized Services for the Prevention and Treatment of Depression" in September 2020, which identified six key tasks: strengthening knowledge and education on prevention and treatment, conducting screening and assessment, improving the capacity for early diagnosis and standardized treatment, increasing intervention efforts for key populations, strengthening the psychosocial hotline service, and conducing timely psychosocial interventions, and noted that healthcare institutions use the PHQ-9 scale to screen for depression and assess of the status of depression^40^.

To this end, this present study collected basic information (gender, age, presence of other serious illnesses, and place of residence) was collected from schistosomiasis populations and control populations in Jiangxi Province, an endemic province in China, and all participants completed the PHQ-9 questionnaire for the study. All the survey administrators received training on the questionnaire. This was a cross-sectional survey designed to assess the occurrence of depression in the schistosomiasis japonica population and to compare the variability of depressive symptoms among different population groups. The type of schistosomiasis epidemic in the area of residence, the degree of epidemiological control, and Per-capita Disposable Income of Rural Households (P-DIRH) and Per-capita Gross Domestic Product (P-GDP) in the area of residence were also collected to explore whether there are potential risks of these factors (including gender and age).

## Methods

### Participants

The participants were selected from the Jiangxi Province which is one of the provinces in China with severe schistosomiasis japonica endemic. It has a population of approximately 5,229,400 people who are at risk of contracting the disease. The province has 5,483 cases of advanced schistosomiasis patients, which is the third highest in the country^41^, including 3,878 AS aged 20–75 years; 24,067 people infected with schistosomiasis and causing damage to the liver damage are registered and counted.

Advanced schistosomiasis population (AS): According to the National Standardized Diagnostic Criteria for Schistosomiasis (WS261-2006), when individuals infected with schistosomes do not receive timely and adequate treatment, typically after 2–10 years of pathological progression, this results in hepatic fibrosis portal hypertension syndrome, severe growth disturbances or significant granulomatous proliferation of the colon.

Non-advanced schistosomiasis population (N-AS): It is a condition in which the liver of a person infected with *Schistosoma* haematobium shows chronic schistosomal liver fibrosis^42^. However, the disease has not yet progressed to the advanced stage.

Control group (CG): Living in schistosomiasis endemic areas, but not infected with schistosomes.

## Research methodology

This study used a non-probability sample survey method, using the advanced schistosomiasis population as the base, and matching the population with chronic schistosomiasis and uninfected schistosomiasis populations with 1.5–2 times the number of people. Matching principles: residence in the same county (try to select the same township), the same gender, and similar age (± 5 years). The survey was conducted between 30 May 2023 and 1 September 2023 using an online questionnaire by the investigators on the online platform “Questionnaire Star” (https://www.wjx.cn/), with mandatory requirements were set for each entry to ensure the completeness of the questionnaire. To be eligible for the study, participants had to meet the following criteria: (1) be willing to participate in the study after being introduced to the background and content of the survey by the investigator; (2) live in the district where the survey was conducted and be in a schistosomiasis-endemic area; (3) be of normal mental capacity, be able to understand the content of the survey items and be able to express themselves clearly; and (4) be aged between 20 and 75 years of age.

## Research tools

The PHQ-9, a diagnostic tool for the common Primary Care Evaluation of Mental Disorders (PRIME-MD), was chosen. It consists of nine items, and respondents indicate whether they have experienced the condition described in the item and how often in the past two weeks. Each item is given a score of 0–3 based on the response (0 = not at all; 1 = several days; 2 = more than half the days; 3 = nearly every day). The total score is calculated by adding the scores of all nine items, with a score range of 0–27. Depression is categorized into 5 levels based on the PHQ-9's scoring system: NONE (0–4 points), MILD (5–9 points), MODERATE (10–14 points), MODERATELY SEVERE (15–19 points), and SEVERE (≥ 20 points)^43^. We used the Chinese version of the PHQ-9. It showed good reliability and validity in adult and adolescent populations in adult and adolescent populations in mainland China and China-Taiwan, with internal consistency scores of 0.854 and 0.84, and test–retest reliability scores of 0.873 and 0.80^44,45^.

### Definition of factors

#### Socioeconomics

The participants in this study were all rural households, so we chose per-capita disposable income of rural households (P-DIRH) and per-capita gross domestic product (P-GDP) as the indicators for this study. The data for each county and district were obtained from the Jiangxi Statistical Yearbook (2022). For this reason, we will use the province’s per capita disposable income of rural residents and per capita GDP as the critical dividing line to compare the indicators of each county and district, and then assign qualitative data, i.e., “high” or “low” respectively.

#### Age

In this study, the age of the participants was categorized into three age groups: 20–44, 45–59, and 60–75 years old^46,47^.

#### Presence of other serious illnesses

These include cardiovascular disease, tumors, diabetes, kidney disease, hepatitis/cirrhosis, and other chronic physical illnesses that have a serious impact on quality of life. We will define “yes” if any of these conditions are present and “no” otherwise.

#### Type of schistosomiasis endemic area^48^

The types of schistosomiasis endemic areas in China include marshland and lake regions, hilly and mountainous regions, and plain regions with waterway networks, and in Jiangxi Province, there are only marshland and lake regions and hilly and mountainous regions.

Marshland and lake regions: Distributed on both sides of the Yangtze River and the large and small lakes connected to the Yangtze River, including Poyang Lake and the inner lakes below the Hukou in Jiangxi Province, which are also the areas with schistosomiasis is currently highly prevalent.

Hilly and mountainous regions: the altitude of the distribution area is different, the low is less than 1 m, the high is up to 2400 m, the distribution of the epidemic area is closely related to the direction of the mountain range, the infected area between the county (city), townships (towns) can be connected to a piece of land, there are independent blocks, the scope of a very small.

Mixed regions: refers to the presence of both marshland and lake regions and hilly and mountainous regions within the same endemic county area.

#### Endemic status of schistosomiasis

According to the National Standardized Control and Elimination of Schistosomiasis (GB 15976–2015), there are currently only 2 types of schistosomiasis epidemiological status in each county in Jiangxi Province, namely, transmission interruption and elimination. Criteria for transmission interruption (which should meet the following items at the same time): (a) no locally infected schistosomiasis patients have been found for 5 consecutive years; (b) locally infected schistosomiasis animals have not been found for 5 consecutive years; (c) infected snails have not been detected for 5 consecutive years; (d) Establish and improve a sensitive and effective monitoring system for schistosomiasis on a district basis. Criteria for Elimination: no locally infected schistosomiasis patients, diseased animals, or infected snails have been detected for five consecutive years after meeting the requirements for transmission interruption.

### Statistical analysis

Statistical analysis was performed using the SPSS Statistics 27.0 software (SPSS Inc., Chicago, IL, USA) with a test level of α = 0.05. The method of data analysis procedure consisted of the following steps:

Descriptive statistics were used for general data and other relevant descriptions. Comparisons of depression rates were tested using the χ^2^ test; given the non-normal distribution of PHQ-9 score levels, score levels were tested using a non-parametric independent samples test.

Univariate analysis of variance between demographic and sociological variables in different populations was performed using the Chi-square test.

Binary logistic regression analysis was used to screen for factors associated with depression, and the final report was presented as ORs (odds ratios), 95% CIs (confidence intervals), and significance levels (P-values). When conducting the analysis, we also took into account that there may be some association between the variables that were not significantly different in the univariate analysis and other confounding variables, and to avoid masking the true effect of the variable being masked by the role of other confounding indicators, all variables with P < 0.20 in the univariate analysis were included in the multivariate analysis, and then the variables were retained in the multivariate in the stepwise backward selection process after P < 0.10 in the multivariate model^49^.

### Ethical considerations

Our research protocol was approved by The Medical Ethics Committee of Jiangxi Provincial Institute of Parasitic Disease. All methods and steps were carried out following the relevant guidelines and regulations of the committee. This study conforms to the provisions of the Declaration of Helsinki (as revised in Seoul, Korea, October 2008).

Informed consent was obtained from all participants for this study. Each participant was informed of the purpose, procedures, and nature of the study during the survey, and participants were given the opportunity to withdraw their data at any time during the study. All participants were anonymous. Any participant whose questionnaire results showed a score of mild depression or higher was advised to go to see a hospital specialist for further diagnosis and treatment.

## Results

### The participants’ general data

A total of 8810 people aged between 20 to 75 years participated in this survey, with a mean age of 60.03 ± 12.30 years. Of these participants, 1840 were AS (20.89%), 3512 were N-AS (39.86%) and 3458 were CG (39.25%). The detailed distribution of social and demographic characteristics is shown in Table 1.

**Table 1:** Socio and demographic characteristics.

Variables/categories	AS		N-AS		CG	
	Number	Proportion (%)	Number	Proportion (%)	Number	Proportion (%)
**Age**						
20–44 years	37	2.01	326	9.28	681	19.69
45–59 years	293	15.92	1045	29.76	1122	32.45
60–75 years	1510	82.07	2141	60.96	1655	47.86
**Gender**						
Male	1114	60.54	1932	55.01	1691	48.90
Female	726	39.46	1580	44.99	1767	51.10
**Presence of other serious illnesses**						
Yes	630	34.24	591	16.83	291	8.42
No	1210	65.76	2921	83.17	3167	91.58
**P-DIRH**						
High	1259	68.42	2301	65.52	1955	56.54
Low	581	31.58	1211	34.48	1503	43.46
**PC-GDP**						
High	462	25.11	675	19.22	600	17.35
Low	1378	74.89	2837	80.78	2858	82.65
**Type of schistosomiasis endemic area**						
Lake-marsh type	342	18.59	725	20.64	670	19.38
Hilly and mountainous regions	764	41.52	1209	34.42	1421	41.09
Mixed regions	734	39.89	1578	44.93	1367	39.53
**Endemic status of schistosomiasis**						
Transmission interruption	1228	66.74	2912	82.92	2403	69.49
Elimination	612	33.26	600	17.08	1055	30.51

### PHQ-9 levels of depression severity, *n* (%)

Overall, AS had the highest occurrence of depression (34.35%), while N-AS and CG had 22.35% and 22.24% respectively, and the occurrence of depression was significantly higher in AS than in N-AS and CG (*χ*_*C*S_^2^ = 89.256, *χ*_*NS*_^*2*^ = 90.540, P < 0.001), whereas the comparison between N-AS and CG was not statistically significant (*χ*^*2*^ = 0.013, P > 0.05). According to Table 2, the incidence of mild depressive symptoms ranged from 61.08% to 75.54% of the occurrence of depression in their respective populations. This suggests that mild depression is prevalent in all types of populations. In terms of PHQ-9 scores, none of the scores were statistically significant, except for the mean score of “moderate”, which was statistically significant in all three populations (H = 22.746, P < 0.001).

**Table 2:** Comparing of the prevalence of depressive symptoms and mean scores levels in different populations.

PHQ-9 levels of depression severity	AS (*N* = 1840)		N-AS (*N* = 3512)		CG (*N* = 3458)	
	%(*n*)	Score (*mean* + *SD*)	%(*n*)	Score (*mean* + *SD*)	%(*n*)	Score (*mean* + *SD*)
None(0-4)	65.65(1208)	0.20 ± 0.48	77.65(2727)	0.13 ± 0.40	77.76(2689)	0.17 ± 0.44
Mild(5-9)	20.98(386)	1.69 ± 1.09	16.88(593)	1.70 ± 1.13	16.11(557)	1.77 ± 1.32
Moderate(10-14)	9.84(181)	6.61 ± 5.24	2.99(105)	5.86 ± 5.11	3.44(119)	8.87 ± 5.28
Moderately severe(15-19)	2.93(54)	15.72 ± 4.15	1.94(68)	15.31 ± 4.73	2.20(76)	15.24 ± 4.84
moderately severe(20-27)	0.60(11)	23.45 ± 2.38	0.54(19)	22.63 ± 2.14	0.49(17)	22.65 ± 2.23

### Depression and its associated factors

It has been shown that using a PHQ-9 score of 10 as a threshold for depressive symptoms has greater clinical significance^50,51^, and for this reason, we used the PHQ-9 ≥ 10 to denote depression when analyzing factors associated with depression.

#### Univariate analysis

In Table 3 shows that the occurrence of depression in the AS population increased with age; it was significantly higher in males than in females; it was significantly higher in the coexistence of AS and other serious diseases than in the AS population alone; the higher the economic level of the individual and the society as a whole, the lower the occurrence of depression; and the occurrence of depression was significantly higher in the lake-marsh type of environmental outbreak than in the other two types of environmental outbreak (*χ*^*2*^ = 57.848 & 143.226, P < 0.001); and there was no association between the level of control of schistosomiasis epidemics and the level of depression.

**Table 3:** Univariable analysis of socio and demographic factors associated with moderate/severe depressive symptoms in advanced schistosomiasis.

Variables	Total *n* = 1840	Depressed *n*(%)	OR(*95%CI*)	*P*-value
**Age**				
20–44 years	37	1(2.7)	1	0.021
45–59 years	293	28(9.56)	3.804(0.502–28.812)	
60–75 years	1510	217(14.37)	6.042(0.824–44.296)	
**Gender**				
Male	1114	175(15.71)	1	< 0.001
Female	726	71(9.78)	0.582(0.434–0.780)	
**Presence of other serious illnesses**				
Yes	630	157(24.92)	1	< 0.001
No	1210	89(7.36)	0.239(0.181–0.317)	
**P-DIRH**				
High	1259	76(6.04)	1	< 0.001
Low	581	170(29.26)	6.438(4.804–8.629)	
**PC-GDP**				
High	462	39(8.44)	1	< 0.001
Low	1378	207(15.02)	1.917(1.338–2.747)	
**Type of schistosomiasis endemic area**				
Marshland and lake regions	342	110(32.16)	1	< 0.001
Hilly and mountainous regions	764	98(12.83)	0.310(0.227–0.423)	
Mixed regions	734	38(5.178)	0.115(0.077–0.171)	
**Endemic status of schistosomiasis**				
Transmission interruption	1228	151(14.02)	1	0.056
Elimination	612	95(18.36)	1.311(0.993–1.729)	

In Table 4 shows that the occurrence of depression in the N-AS population seems to be higher only in the elderly, and the comparison of depression rates between the two age groups below 60 years is not statistically significant (*χ*^*2*^ = 2.022, P > 0.05); females are higher than males; and the significance of the coexistence of N-AS and other serious diseases is higher than that of the N-AS population alone; the higher the economic level of the individual and of the whole society, the lower the occurrence of depression; the occurrence of depression was significantly higher in the lake-marsh and hill-type environmental outbreaks than in the mixed environmental type outbreaks (*χ*^*2*^ = 24.599 & 25.744, P < 0.001), although the comparison between them was not statistically significant, and the occurrence of depression was lower in the areas of transmission interruption areas than in elimination areas.

**Table 4:** Univariable analysis socio and demographic factors associated with moderate/severe depressive symptoms in non-advanced schistosomiasis.

Variables	Total *n* = 1840	Depressed *n*(%)	OR(*95%CI*)	*P*-value
**Age**				
20–44 years	326	13(3.99)	1	< 0.001
45–59 years	1045	26(2.49)	0.614(0.312–1.210)	
60–75 years	2141	153(7.15)	1.853(1.039–3.305)	
**Gender**				
Male	1932	92(4.76)	1	0.043
Female	1580	100(6.33)	1.351(1.010–1.808)	
**Presence of other serious illnesses**				
Yes	591	105(17.77)	1	< 0.001
No	2921	87(2.98)	0.142(0.105–0.192)	
**P-DIRH**				
High	2301	66(2.87)	1	< 0.001
Low	1211	126(10.40)	3.933(2.894–5.343)	
**PC-GDP**				
High	675	13(1.93)	1	< 0.001
Low	2837	179(6.31)	3.429(1.941–6.060)	
**Type of schistosomiasis endemic area**				
Marshland and lake regions	725	63(8.69)	1	< 0.001
Hilly and mountainous regions	1209	99(8.19)	0.937(0.674–1.304)	
Mixed regions	1578	30(1.90)	0.204(0.131–0.318)	
**Endemic status of schistosomiasis**				
Transmission interruption	2912	102(3.50)	1	< 0.001
Elimination	600	90(15.00)	4.862(3.606–6.554)	

The occurrence of depression in the CG population is shown in Table 5 to be at two extremes, with significantly higher rates of depression between the two age groups of under 45 and under 60 years than between the ages of 45–59 years (*χ*^*2*^ = 17.481 & 36.564, *P* < 0.001), while the comparisons between them were not statistically significant; the rates of depression in females are slightly higher than in males, but the difference is not statistically significant; the coexistence of CG and other serious diseases was more significant in the CG population than in the CG population alone; the higher the economic level of the individual and of the total society as a whole, the lower the occurrence of depression; the occurrence of depression in the lake-marsh type of environmental outbreaks was significantly higher than that in the other two environmental outbreaks (*χ*^*2*^ = 37.327 & 17.347, P < 0.001); and there was no association between the level of control of schistosomiasis epidemics and the high occurrence of depression.

**Table 5:** Univariable analysis socio and demographic factors associated with moderate/severe depressive symptoms in control group.

Variables	Total *n* = 1840	Depressed *n*(%)	OR(*95%CI*)	*P*-value
**Age**				
20–44 years	681	46(6.75)	1	
45–59 years	1122	30(2.67)	0.379(0.237–0.607)	< 0.001
60–75 years	1655	136(8.22)	1.236(0.874–1.748)	
**Gender**				
Male	1691	96(5.68)	1	0.277
Female	1767	116(6.56)	1.167(0.883–1.543)	
**Presence of other serious illnesses**				
Yes	291	42(14.43)	1	< 0.001
No	3167	170(5.37)	0.336(0.234–0.483)	
**P-DIRH**				
High	1955	48(2.46)	1	< 0.001
Low	1503	164(10.91)	4.866(3.501–6.763)	
**PC-GDP**				
High	600	6(1.00)	1	< 0.001
Low	2858	206(7.21)	7.690(3.399–17.401)	
**Type of schistosomiasis endemic area**				
Marshland and lake regions	670	74(11.04)	1	
Hilly and mountainous regions	1421	58(4.08)	0.343(0.240–0.490)	< 0.001
Mixed regions	1367	80(5.85)	0.501(0.360–0.697)	
**Endemic status of schistosomiasis**				
Transmission interruption	2403	154(6.41)	1	0.304
Elimination	1055	58(5.50)	0.850(0.622–1.160)	

#### Multivariable analysis

Based on the multivariate analysis in Table 6, moderate to major depressive symptoms in the AS group were significantly associated with several factors. These factors included being over 60 years of age, being male, having comorbidities with other serious illnesses, economic hardship, and living in endemic areas of lakes and marshes. However, the level of the society’s economy and the degree of control of schistosomiasis epidemics were not linked to the occurrence of moderate to major depression; In the N-AS group, moderate to major depressive symptoms were significantly associated with comorbidities with other serious diseases, personal economic difficulties, lake-marsh endemic areas, and the level of control of schistosomiasis epidemics. Age, gender, and economic level of the society did not show any association with the occurrence of moderate to major depression. Whereas, in the CG group, the occurrence of moderate to major depressive symptoms was significantly associated with the young and the old, the comorbidities of other serious diseases, personal economic difficulties, the economic low of the society, and the lake-marsh endemic areas.

**Table 6:** Multivariable analysis of factors associated with moderate/severe depressive symptoms.

Variables	AS		N-AS		CG	
	OR(*95%CI*)	*P*-value	OR(*95%CI*)	*P*-value	OR(*95%CI*)	*P*-value
**Age**						
20–44 years	1	0.004	1	0.109	1	< 0.001
45–59 years	3.612 (0.434–30.077)		0.522 (0.252–1.079)		0.307 (0.188–0.501)	
60–75 years	7.172 (0.892–57.655)		0.810 (0.419–1.566)		0.943 (0.651–1.367)	
**Gender**						
Male	1	< 0.001	1	0.477	–	
Female	0.537 (0.386–0.748)		1.127 (0.811–1.565)			
**Presence of other serious illnesses**						
Yes	1	< 0.001	1	< 0.001	1	< 0.001
No	0.310 (0.225–0.427)		0.177 (0.128–0.244)		0.365 (0.246–0.541)	
**P-DIRH**						
High	1	< 0.001	1	< 0.001	1	< 0.001
Low	5.233 (3.428–7.988)		3.161 (2.135–4.680)		2.500 (1.722–3.629)	
**PC-GDP**						
High	1	0.059	1	0.055	1	< 0.001
Low	0.583 (0.333–1.020)		2.007 (0.985–4.091)		7.282 (2.970–17.856)	
**Type of schistosomiasis endemic area**						
Marshland and lake regions	1	< 0.001	1	< 0.001	1	< 0.001
Hilly and mountainous regions	0.373 (0.175–0.793)		0.391 (0.191–0.799)		0.242 (0.162–0.362)	
Mixed regions	0.163 (0.106–0.251)		0.259 (0.162–0.415)		0.430 (0.300–0.615)	
**Endemic status of schistosomiasis**						
Transmission interruption	1	0.826	1	< 0.001	–	
Elimination	1.084 (0.529–2.217)		3.818 (1.956–7.452)			

## Discussion

Depression is a common mental illness that poses a health risk to an individual’s mind, emotions, and body due to low mood, loss of energy, and sadness^52^. The PHQ-9 has been used to detect symptoms of depression in various populations beyond its original use in primary care settings. In particular, the DSM-5 (Diagnostic and Statistical Manual of Mental Disorders, Fifth Edition) (https://www.psychiatry.org/dsm5) also recommends the use of the PHQ-9 to assess the severity of depression^35^.

AS japonica can be considered an extreme form of chronic schistosomiasis japonica, more severe than the liver and spleen disease of late *Schistosoma mansoni* infection found in Africa and the Americas^21^. AS japonica is a chronic and disabling disease associated with portal hypertension, splenomegaly, ascites, and gastroesophageal variceal bleeding, or with severe growth retardation or granulomatous disease of the large intestine. In China, advanced cases are registered and managed separately from patients with general chronic schistosomiasis.

However, there are few studies on the occurrence of depression in vulnerable groups such as schistosomiasis sufferers, especially in N-AS populations, and there is a need to fill the existing gaps in the literature. In this context, our study investigated the sample characteristics of a PHQ-9-based psychometric measure in a schistosomiasis-endemic area in China.

In this study, the occurrence of depression in AS was 34.35%, which was higher than that of N-AS (22.35%) and CG (22.24%), using the Community Generalized Depression Screening Tool, PHQ-9, to screen patients with depression in different types of populations in schistosomiasis-affected areas. It shows that AS patients are more likely to develop depressive disorders than the general population/mild schistosomiasis patients due to the prolonged course of the disease, numerous and severe complications, low cure rate, and the need for long-term and repeated treatment^53,54^. Meanwhile, our survey also showed that the occurrence of depression in both types of population was much higher than in other non-schistosomiasis-endemic rural areas^12–18^.

However, the occurrence of AS depression in our study was much lower than other currently reported rates of AS depression (41.7%-82.2%)^55–59^, which may be due to the difference in the focus groups, as the target populations of the previously reported rates of AS depression were all focused clinical samples, which themselves had already had more severe physical and psychological consequences due to the presence of multiple comorbidities. However, our results also showed a 54.6% occurrence of depressive symptoms when AS was comorbid with other serious illnesses.

The sensitivity and specificity of the PHQ-9 questionnaire for detecting major depression in the general population is 88%^60^. AS the diagnosis of depression in this study was self-reported, it was not possible to calculate the sensitivity and specificity of the PHQ-9. However, among the schistosomiasis patients (AS & N-AS) screened for depression, 18.29% had a PHQ-9 ≥ 5 (with some depressive symptoms) and 8.18% had a PHQ-9 ≥ 10 (with moderate/severe depressive symptoms).

In areas where schistosomiasis is endemic, we conducted various forms of health education. The main aim was to raise awareness that schistosomiasis is preventable and curable. The target groups were mainly community members and school children. Most of the groups that participated were N-AS^61–64^. This may explain the lack of difference in the occurrence of depression between N-AS and CG. Schistosomiasis control work in the elimination areas relies mainly on surveillance, and related health education has declined. This has led to a higher occurrence of AS and N-AS depression in the elimination areas compared to the transmission interruption areas.

The gender findings of this study are consistent with other studies that have shown a higher prevalence of depression in men with AS than in women^65–67^. This may be due to the fact that men are usually the main breadwinners in the family, and their illness leads to poverty as a result of limited financial resources for the family, resulting in greater psychological distress than in women. The higher prevalence of depression in the older age group may be related to the loss of confidence in life due to the decline in physical fitness, self-care skills and long-term recovery.

Combining the results of the multifactorial analysis, we believe that the causes of depression may be the following: (i) psychological changes brought about by the residents’ schistosome infection and excessive worry about their own health status; (ii) related to the increased worry about their own health status after the improvement of the residents’ lives; (iii) villagers living in the infected area, who cannot live without contact with the water source, increase the nervousness about the possibility of contracting schistosomiasis, especially in the lake and swamp infected area; (iv) the economic base and socio-economic level of the individual determines the occurrence of depression; and (v) the implementation of health education can further reduce the occurrence of depression.

As depressed patients are characterized by more negative automatic thoughts than normal, without cognitive intervention, patients’ negative cognitions will still persist, and even with maintenance antidepressant therapy, its efficacy will not be sustained^68,69^. Psychological interventions given to AS with depression during repeated hospitalizations have been shown to result in a significantly lower percentage of moderate/severe depression at 4 weeks than before the intervention^70^. Through psychological care, we can understand the psychological state of patients in time, eliminate bad emotions, and keep patients in a comfortable mood; through disease knowledge education, patients can actively cooperate with the treatment and adhere to the treatment; through health education, we can urge patients to develop proper dietary habits and healthy lifestyles, thus reducing the high-risk factors that cause the exacerbation of the disease; and the post-discharge follow-up visit can enlist the support of the family and the society, so that the family members can play a role of supervision, support and encouragement, helping patients to establish correct health concepts and change bad life habits, and through the participation of the family and society, the treatment of patients can be strongly guaranteed, the patients’ pain can be reduced, and their quality of life can be improved and enhanced.

### Limitations

There are also some limitations to this study. Firstly, the sample came from only one Chinese province where schistosomiasis is endemic; secondly, due to the short duration of the survey and the fact that the participants were anonymous, it was not possible to repeat the test; and finally, this study did not take much account of other factors that affect depression (e.g. HBV or severe chronic illness). However, our findings are preliminary at this stage. Further research is therefore needed to confirm our findings.

## Conclusion

In conclusion, depression is still relatively common in schistosomiasis patients. The aim of treating depression is to diagnose it as early as possible, standardize the treatment in time, control the symptoms, improve the clinical cure rate, minimize the rate of disability and suicide, prevent relapse and promote the recovery of social function. Therefore, the involvement of mental health workers in the prevention and treatment of schistosomiasis is beneficial. The government and relevant departments should include mental health care in the prevention and treatment of schistosomiasis in the infected areas in order effectively protect the physical and mental health of schistosomiasis patients and residents in the infected areas.

## Data Availability

The datasets analyzed in the current study are available from the corresponding author on reasonable request.
